# Temporal Trends and Burden of Rheumatic Heart Disease in South Asia: A Comprehensive Analysis of Three Decades from Global Burden of Disease Study

**DOI:** 10.5334/gh.1336

**Published:** 2024-06-26

**Authors:** Khalid Naseeb, Muhammad Nauman Khan, Najia Aslam Soomro, Abdul Hameed, Gian Chand, Jaghat Ram, Ahmed Raheem

**Affiliations:** 1 National Institute of Cardiovascular Diseases (NICVD), Karachi, Pakistan; 2 Liaquat National hospital and Medical College, Karachi, Pakistan; 3 Aga Khan University Hospital, Karachi, Pakistan

**Keywords:** South Asia, rheumatic heart disease, burden, prevalence, deaths

## Abstract

**Background:**

The objective of this study is to conduct a temporal analysis of rheumatic heart disease (RHD) disease burden trends over a 30-year period (1991 to 2021), focusing on prevalence, deaths, and disability-adjusted life years (DALYs) in the South Asia (SA).

**Methods:**

In this ecological study, we analyzed data regarding burden of RHD from the Global Burden of Diseases (GBD) study spanning the years 1991 to 2021 for the SA Region. Estimates of the number RHD-related prevalence, deaths, and DALYs along with age-standardized rates (ASR) per 100,000 population and 95% uncertainty intervals (UI) were evaluated.

**Results:**

The overall prevalent cases of RHD in the 2021 were 54785.1 × 10^3^ (43328.4 × 10^3^ to 67605.5 × 10^3^), out of which 14378.8 × 10^3^ (11206.9 × 10^3^ to 18056.9 × 10^3^) were from SA. The ASR of point prevalence showed upward trend between 1991 and 2021, at global level and for SA with an average annual percentage change (AAPC) of 0.40 (0.39 to 0.40) and 0.12 (0.11 to 0.13), respectively. The overall number of RHD-related deaths in the 2021 were 373.3 × 10^3^ (324.1 × 10^3^ to 444.8 × 10^3^), out of which 215 × 10^3^ (176.9 × 10^3^ to 287.8 × 10^3^) were from SA, representing 57.6% of the global deaths. The ASR of deaths also showed downward trend between 1991 and 2021, at global level and for SA with an AAPC of –2.66 (–2.70 to –2.63) and –2.07 (–2.14 to –2.00), respectively. The ASR of DALYs showed downward trend between 1990 and 2019, at global level and for South Asian region with an AAPC of –2.47 (–2.49 to –2.44) and –2.22 (–2.27 to –2.17), respectively.

**Conclusion:**

The rising age-standardized prevalence of RHD remains a global concern, especially in South Asia which contribute to over 50% of global RHD-related deaths. Encouragingly, declining trends in RHD-related deaths and DALYs hint at progress in RHD management and treatment on both a global and regional scale.

## Introduction

Rheumatic heart disease (RHD), a preventable consequence stemming from acute rheumatic fever (ARF), disproportionately affects the world’s most vulnerable populations. In the past few decades, improved living conditions, expanded healthcare access, and the widespread use of penicillin-like medications have significantly reduced the RHD burden, virtually eradicating it from high-income societies [1,2]. However, RHD remains closely linked with poverty, and even in the twenty-first century, it continues to be a significant contributor to annual morbidity and mortality in low- and middle-income countries [3,4]. Annually, RHD is estimated to cause approximately 306 thousand deaths, impacting more than 40.5 million individuals globally [5]. Despite notable advancements in our understanding of RHD and its underlying mechanisms, progress in preventing the disease has been limited. In the absence of an effective vaccine, the primary strategies for RHD control have centered on surgery and penicillin-based treatments. However, the adoption of echocardiographic algorithms for screening has substantially enhanced the accuracy of RHD diagnosis [6].

A fundamental prerequisite for the development of any effective preventive policy for RHD is to accurately ascertain the true disease burden initially [7]. A systematic review conducted by Tibazarwa et al. [8] has unveiled a concerning scarcity of high-quality, population-based prospective studies investigating the incidence of acute rheumatic fever (ARF) worldwide. Surprisingly, population-based studies have been carried out in only 10 out of more than 190 countries globally, with the most recent one concluding over a decade ago. This glaring gap underscores the urgent need for reliable and robust data regarding ARF incidence. Such information is crucial as it forms the foundation for shaping health policies, allocating resources, and guiding clinical management, especially in low- and middle-income nations.

The population of the South Asian region, primarily encompassing Bangladesh, Bhutan, India, Nepal, and Pakistan, faces an elevated risk of cardiovascular diseases (CVD) [9,10,11]. This heightened risk is attributable to various economic, environmental, and genetic factors [12,13]. The increasing burden of CVD, particularly RHD, places additional economic and managerial pressures on these countries, which are already grappling with economic challenges. Additionally, the South Asian region comprises nearly 1/4 of the total world population and increasing burden of RHD in these region has a significant impact on the global sequels of the RHD. Recognizing regional dynamics is equally crucial for crafting effective policies tailored to the specific requirements and challenges of this region. Poverty is a key driver of the persistent prevalence of RHD in developing and underdeveloped nations. Factors such as overcrowding, substandard living conditions, and the absence of organized community-based screening and surveillance systems for rheumatic fever and pharyngitis exacerbate the disease burden [14].

Furthermore, the accurate estimation of disease burden remains a challenge due to the scarcity of high-quality, reliable epidemiological studies in these regions. To effectively address the mounting burden of RHD in this region, gaining a deeper understanding of underlying disease patterns and establishing dependable projections is vital. Therefore, the objective of this study was to conduct a temporal analysis of trends in RHD disease burden spanning 30 years (1991 to 2021), focusing on prevalence, mortality, and disability-adjusted life years (DALYs) at global level, regional level, and country level within the South Asian region from the Global Burden of Diseases (GBD) study.

## Material and Methods

The GBD framework offers a comprehensive methodological and conceptual basis for gauging health-related losses worldwide. This framework serves as a valuable tool for assessing the progress made and challenges faced in controlling diseases globally. In this study, we primarily focused on assessing the burden of RHD by considering its DALYs.

We sourced our data from the Global Health website, specifically from the GBD results tool, which can be accessed at http://ghdx.healthdata.org/gbd-results-tool. The dataset we extracted included estimates of the number of cases and age-standardized rates (ASR) per 100,000 population, along with 95% uncertainty intervals (UI) for these measures spanning the years 1991 to 2021. For a comprehensive understanding of the methodology used for statistical estimation by the Global Burden of Diseases (GBD) study, please refer to the full methodological details reported elsewhere [15].

Our geographical scope included both the global and regional perspective and a specific focus on the South Asian Region, which comprises five countries: Bangladesh, Bhutan, India, Nepal, and Pakistan. Additionally, we segregated the data by gender for the South Asian region.

For trend analysis, we conducted joinpoint regression analysis using the Joinpoint Regression Program, Version 5.0.2, developed by the Statistical Research and Applications Branch of the National Cancer Institute [16]. In our analysis, we employed a log-transformed model, assuming homoscedasticity and uncorrelated error terms. The maximum number of joinpoints were set to the recommended five joinpoints and model selection criteria was data driven weight BIC (Bayesian Information Criterion).

To provide a comprehensive overview of trends, we calculated the average annual percentage change (AAPC) and its corresponding 95% confidence interval (CI) for the entire study period (1991 to 2021), each segment within the final selected model, and three customized time ranges representing the last three decades: 1991 to 2001, 2002 to 2011, and 2012 to 2021.

## Results

**Figure 1:**
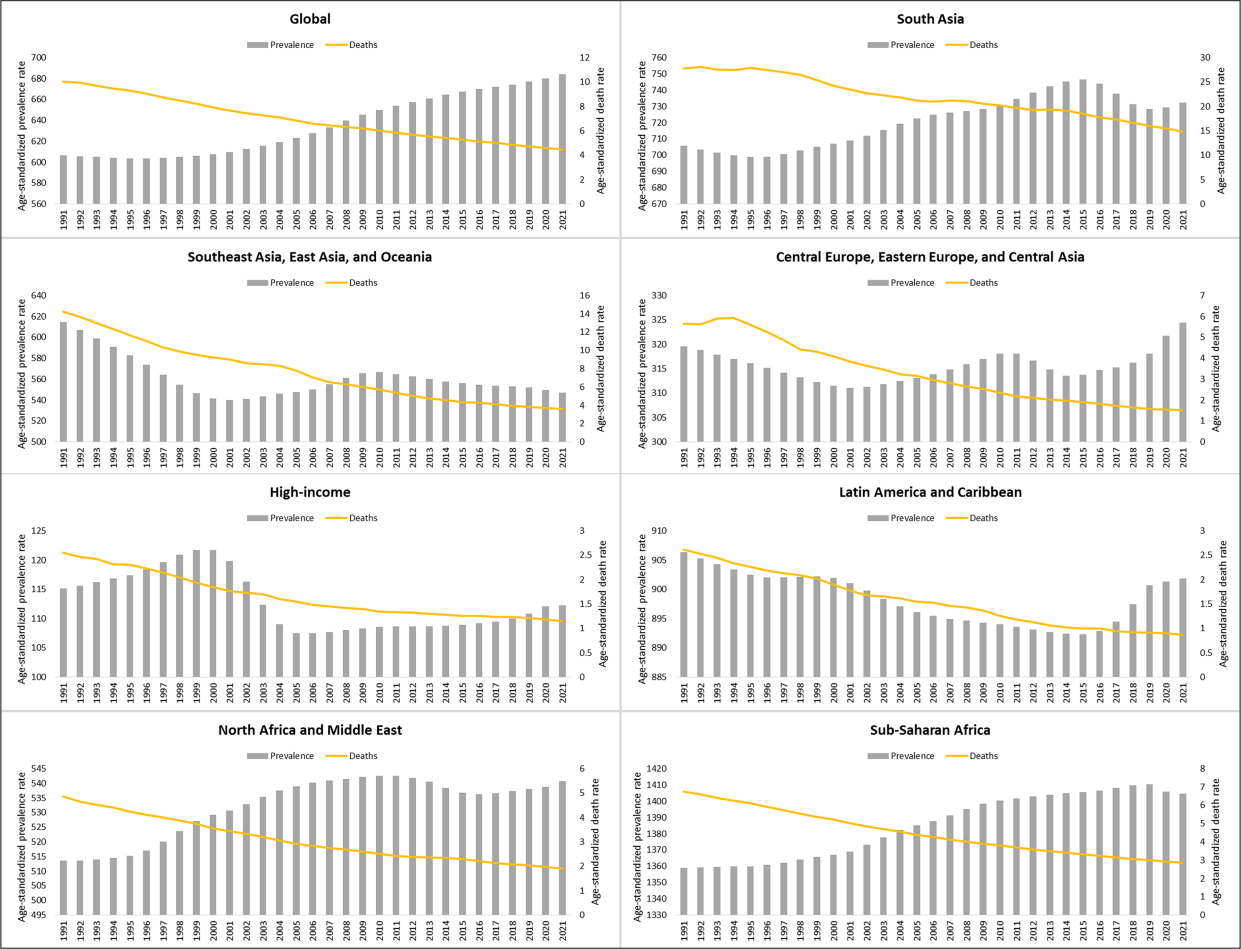
Trend of rheumatic heart disease age-standardized rates of prevalence and deaths at global and regional level between 1991 and 2021.

According to GBD estimates, in the 2021, there were 54785.1 × 10^3^ (95% UI: 43328.4 × 10^3^ to 67605.5 × 10^3^) prevalent cases of RHD globally, with an age-standardized point prevalence of 684.2 per 100,000 population (95% UI: 540.4 to 848.9). The overall age-standardized rate (ASR) showed an upward trend between 1991 and 2021, with an average annual percentage change (AAPC) of 0.40 (95% CI: 0.39 to 0.40) (Table 1, Figure 1).

**Table 1:** Trend of prevalent cases of rheumatic heart disease and age-standardized rate (ASR) per 100,000 from 1991 to 2021 by global, regions, and countries of South Asian region.


	1991		2001		2011		2021		AAPC BETWEEN 1991 AND 2021 (95% CI)
			
	NUMBER × 10^3^ (95% UI)	ASR/100,000 (95% UI)	NUMBER × 10^3^ (95% UI)	ASR/100,000 (95% UI)	NUMBER × 10^3^ (95% UI)	ASR/100,000 (95% UI)	NUMBER × 10^3^ (95% UI)	ASR/100,000 (95% UI)	

**Prevalence**									

Global	32812.6 (26019–40634.8)	606.7 (487–742.4)	38419.8 (30508.8–47404.4)	609.7 (487.9–745.3)	47281.6 (37289.7–58609.1)	653.7 (517.7–807.3)	54785.1 (43328.4–67605.5)	684.2 (540.4–848.9)	0.4 (0.39–0.4)

**By Region**									

South Asia	7465.1 (5848–9294.9)	705.8 (559.5–878.5)	9316.3 (7339.7–11663.5)	708.9 (562.6–878.4)	12184.2 (9448.9–15338.6)	734.4 (574.1–916.4)	14378.8 (11206.9–18056.9)	732.2 (575.8–916.5)	0.12 (0.11–0.13)

Southeast Asia, East Asia, and Oceania	11026.6 (8604.5–13678.3)	614.7 (489.1–755.4)	10898.1 (8517.2–13373)	540.1 (427.6–657.7)	12411.8 (9953.3–15104.1)	564.7 (449.7–689)	12461 (10102.7–15051.4)	546.9 (435.5–670.8)	–0.39 (–0.4 – –0.37)

Central Europe, Eastern Europe, and Central Asia	1453.4 (1304.1–1633.7)	319.5 (285.7–361.1)	1446.4 (1289.9–1643.8)	311 (274.4–355.8)	1481.5 (1304–1700.9)	318.1 (275.2–371.7)	1487.7 (1296.9–1713.9)	324.4 (275.1–384.2)	0.05 (0.04–0.05)

High-income	1277.9 (1143.7–1440.5)	115.1 (103.1–129.1)	1539 (1383–1713.7)	119.9 (107.6–133.4)	1549.5 (1439.9–1685)	108.7 (98.7–120.6)	1826.2 (1659.1–2016.9)	112.3 (100.6–127)	–0.07 (–0.1 – –0.04)

Latin America and Caribbean	3584.1 (2795.7–4506.6)	906.4 (719.1–1122.6)	4358.8 (3421.1–5491.9)	901 (714.9–1116.2)	5016.7 (3920.2–6244.8)	893.5 (703.4–1107.6)	5598 (4453.4–6924.1)	901.8 (717.7–1115.9)	–0.02 (–0.02 – –0.01)

North Africa and Middle East	1668.6 (1296.4–2077.2)	513.5 (407.4–630.7)	2280.4 (1772.7–2852.1)	530.8 (420.7–652.5)	2979.4 (2318.7–3708.5)	542.5 (426.8–668.8)	3508.1 (2763.1–4300.6)	540.8 (428.6–660.4)	0.17 (0.16–0.17)

Sub-Saharan Africa	6336.8 (4870.3–7985.1)	1358.9 (1072.6–1692.3)	8580.7 (6566.5–10862.9)	1369.1 (1086.1–1709.5)	11658.5 (8989.8–14739.2)	1401.7 (1108.4–1741.5)	15525.2 (11847.4–19638.7)	1404.4 (1109.4–1758.6)	0.11 (0.11–0.11)

**By Country**									

India	5756.6 (4515.4–7196.4)	685.9 (544.3–855.3)	6905.3 (5373.1–8618)	669.9 (526–831.8)	9193.4 (7112.2–11604.6)	706.8 (550.3–882.8)	10505.2 (8167.6–13207.3)	690.9 (539.6–867.3)	0.03 (0.02–0.04)

Pakistan	885.9 (685.5–1109.1)	878.2 (695–1086.4)	1346.6 (1038.1–1709.1)	1002.4 (789.4–1255.7)	1705.8 (1322.4–2154.2)	958.4 (758.6–1194.5)	2340.9 (1835.9–2921.7)	991 (789.8–1236)	0.42 (0.39–0.44)

Bangladesh	700.2 (556.6–880.7)	710.5 (569–873.1)	909 (718.4–1142.4)	721.1 (569.6–908.1)	1084.1 (837.6–1377)	723.7 (566.1–910.4)	1303.9 (1013.7–1622.5)	755.6 (588.8–937)	0.21 (0.2–0.21)

Nepal	118.1 (91.5–149.3)	666.9 (524.3–834.4)	150.8 (119–190.1)	671 (530.7–843)	195.4 (151.3–244.4)	712.7 (558.5–885.9)	222.7 (173.4–283.3)	692.5 (543.5–875.1)	0.12 (0.1–0.13)

Bhutan	4.4 (3.4–5.4)	721.2 (566.6–890.2)	4.6 (3.5–5.9)	724.3 (564.6–912.4)	5.5 (4.2–6.9)	733.2 (573.5–912.1)	6.1 (4.8–7.7)	733.2 (576.9–916.4)	0.05 (0.05–0.06)


UI: uncertainty interval, CI: confidence interval, AAPC: average annual percentage change.

Similarly, for South Asian region of GBD study, in the 2021, there were 14378.8 × 10^3^ (95% UI: 11206.9 × 10^3^ to 18056.9 × 10^3^) prevalent cases of RHD, with an age-standardized point prevalence of 732.2 per 100,000 population (95% UI: 415.79 to 916.5). The ASR for South Asian region also showed an upward trend between 1991 and 2021, with an AAPC of 0.12 (95% CI: 0.11 to 0.13) (Table 1, Figure 1). A similar, significant upward trends were observed at gender level and country level analysis of South Asian region (Table 1).

In the 2021, RHD accounted for 13426.4 × 10^3^ (95% UI: 11517 × 10^3^ to 15780.2 × 10^3^) DALYs globally and 7656.5 × 10^3^ (95% UI: 6440.1 × 10^3^ to 9619.4 × 10^3^) DALYs in the South Asian region, with an ASR of 162.1 per 100,000 population (95% UI: 139.1 to 190.5) and 453.6 per 100,000 population (95% UI: 380.1 to 580.9) at global level and South Asian region, respectively (Table 2, Figure 1). The ASR of DALYs showed downward trend between 1991 and 2021, at global level and for South Asian region with an AAPC of –2.47 (95% CI: –2.49 to –2.44) and –2.22 (95% CI: –2.27 to –2.17), respectively.

**Table 2:** Trend of DALYs (Disability-Adjusted Life Years) of rheumatic heart disease and age-standardized rate (ASR) per 100,000 from 1991 to 2021 by global, regions, and countries of South Asian region.


	1991		2001		2011		2021		AAPC between 1991 and 2021 (95% CI)
			
	NUMBER × 10^3^ (95% UI)	ASR/100,000 (95% UI)	NUMBER × 10^3^ (95% UI)	ASR/100,000 (95% UI)	NUMBER × 10^3^ (95% UI)	ASR/100,000 (95% UI)	NUMBER × 10^3^ (95% UI)	ASR/100,000 (95% UI)	

**DALYs (Disability-Adjusted Life Years)**									

Global	16305.4 (13720–19250.4)	341.6 (287.7–403)	15202.1 (13014–17989.2)	265 (226.7–313.7)	14284.9 (12356.5–17211)	205.4 (177.6–247.8)	13426.4 (11517–15780.2)	162.1 (139.1–190.5)	–2.47 (–2.49 – –2.44)

**By Region**									

South Asia	7314.8 (5818.2–9648.2)	880.7 (687.3–1184)	7956.4 (6305.5–10289.2)	752.1 (581.9–994.7)	8230.7 (6688.3–10644.7)	611.5 (488.3–809.2)	7656.5 (6440.1–9619.4)	453.6 (380.1–580.9)	–2.22 (–2.27 – –2.17)

Southeast Asia, East Asia, and Oceania	5158.6 (4340.6–5881.4)	383.4 (324.2–438.5)	3897.4 (3454.5–4411.3)	236.5 (211.8–266.9)	3016.8 (2677.1–3373)	147.5 (131.7–164.2)	2655.4 (2234.5–3145.5)	106.2 (89.4–125.8)	–4.22 (–4.26 – –4.17)

Central Europe, Eastern Europe, and Central Asia	952 (913–1009)	202.7 (194.3–214.8)	681.5 (649.6–721.5)	138.7 (132–147.1)	415.8 (388.6–450.5)	81.3 (75.4–88.6)	316.6 (284.6–356.5)	58.8 (51.9–67.1)	–4.01 (–4.09 – –3.94)

High-income	706.9 (668.1–738.9)	60.6 (57.4–63.4)	556.5 (516.2–592.5)	39.7 (36.9–42.5)	499.1 (447.5–539)	29 (26.3–31.5)	532.3 (471.9–583.8)	25.8 (23–28.6)	–2.8 (–2.85 – –2.75)

Latin America and Caribbean	467.8 (398.6–559)	134.7 (117.4–156.9)	456 (373.7–571.3)	103.3 (86.5–126.6)	440.2 (345.3–568.9)	81.7 (65–104.4)	445.4 (343.4–591.5)	71.5 (55–95.3)	–2.1 (–2.13 – –2.05)

North Africa and Middle East	698.5 (513.3–904.5)	213.2 (154.8–274.8)	595.9 (476.1–737.1)	152.2 (120.7–185.4)	543.6 (457.7–651.3)	111.1 (94.8–132)	511.3 (414.4–619.2)	86.8 (71.3–104.2)	–2.94 (–2.97 – –2.91)

Sub-Saharan Africa	1006.9 (805.8–1248.3)	264.5 (213.3–320.6)	1058.3 (847.9–1305)	211.5 (172.4–257.6)	1138.8 (898.9–1473.3)	170.9 (139–213.4)	1308.8 (984.2–1741.5)	145.3 (114.3–186.8)	–1.98 (–1.99 – –1.97)

**By Country**									

India	5868.2 (4662.4–7706.9)	884 (688.9–1186.9)	6318.1 (5041.4–8147)	741.9 (582–976.3)	6462 (5244.1–8413.1)	602.7 (478.8–801.3)	5745.5 (4785.7–7177.7)	431.5 (361.6–548.6)	–2.38 (–2.42 – –2.34)

Pakistan	663.2 (498.4–923.2)	830.3 (617.7–1186.2)	903.3 (684.1–1221.9)	889.5 (665.3–1232.1)	986.5 (752.6–1316.7)	717.6 (543.6–992)	1088.9 (830.1–1467.7)	585 (443.3–816.9)	–1.16 (–1.19 – –1.13)

Bangladesh	639.5 (452.7–968.1)	857.9 (575.4–1329.6)	608.4 (425.9–949.9)	706.9 (485.3–1117.6)	653 (472.9–1001.3)	574.9 (414–895.3)	688.5 (490.1–1115.2)	457.6 (325–745.6)	–2.08 (–2.15 – –2.02)

Nepal	140 (97.7–208.6)	990.6 (666.5–1559.3)	123.2 (84.8–182.2)	703.1 (468.6–1088.8)	126.3 (91.6–190.2)	571.1 (405–876.9)	130.8 (92.1–190.9)	489.9 (343.9–726.5)	–2.31 (–2.34 – –2.27)

Bhutan	3.9 (2.3–6.3)	927.2 (583.5–1559.6)	3.3 (2.3–5.6)	699.8 (469.2–1243.9)	3 (2–5.5)	516.9 (339.9–1007)	2.8 (1.8–4.9)	401.9 (251.2–725.1)	–2.77 (–2.83 – –2.68)


UI: uncertainty interval, CI: confidence interval, AAPC: average annual percentage change.

The overall number of deaths caused by RHD in the 2021 were 373.3 × 10^3^ (95% UI: 324.1 × 10^3^ to 444.8 × 10^3^), out of which 215 × 10^3^ (95% UI: 176.9 × 10^3^ to 287.8 × 10^3^) were from South Asian Region, representing 57.6% of the global deaths (Table 3, Figure 1). The ASR of deaths also showed downward trend between 1991 and 2021, at global level and for South Asian region with an AAPC of –2.66 (95% CI: –2.70 to –2.63) and –2.07 (95% CI: –2.14 to –2.00), respectively. A similar, significant upward trends were observed at gender level and country level analysis of South Asian region (Table 3).

**Table 3:** Trend of deaths due to rheumatic heart disease and age-standardized rate (ASR) per 100,000 from 1991 to 2021 by global, regions, and countries of South Asian region.


	1991		2001		2011		2021		AAPC between 1991 and 2021 (95% CI)
			
	NUMBER × 10^3^ (95% UI)	ASR/100,000 (95% UI)	NUMBER × 10^3^ (95% UI)	ASR/100,000 (95% UI)	NUMBER × 10^3^ (95% UI)	ASR/100,000 (95% UI)	NUMBER × 10^3^ (95% UI)	ASR/100,000 (95% UI)	

**Deaths**									

Global	420.6 (354.4–500.1)	10 (8.5–11.9)	394.8 (335.1–466)	7.7 (6.5–9)	381.7 (325.2–469.1)	5.8 (5–7.2)	373.3 (324.1–444.8)	4.5 (3.9–5.3)	–2.66 (–2.7 – –2.63)

**By Region**									

South Asia	177.9 (137.5–244)	27.7 (21.2–38.3)	196.9 (148.5–265)	23.4 (17.5–32.1)	218.6 (166.8–302.4)	19.7 (14.8–27.7)	215 (176.9–287.8)	14.9 (12.2–20)	–2.07 (–2.14 – –2)

Southeast Asia, East Asia, and Oceania	149 (124.8–172.1)	14.2 (12–16.5)	119.1 (107.3–134)	9 (8.1–10.1)	94.7 (85.9–103.2)	5.4 (4.8–5.9)	89.6 (73–111.1)	3.6 (2.9–4.5)	–4.54 (–4.59 – –4.49)

Central Europe, Eastern Europe, and Central Asia	26.9 (26–28.7)	5.7 (5.5–6)	19.4 (18.9–20.4)	3.8 (3.7–4)	12 (11.5–12.3)	2.2 (2.1–2.2)	9.4 (8.7–10.2)	1.5 (1.4–1.6)	–4.25 (–4.35 – –4.15)

High-income	31.2 (28.7–32.5)	2.5 (2.3–2.7)	27.2 (24.1–28.8)	1.8 (1.6–1.9)	27.1 (23–29.2)	1.3 (1.1–1.4)	30.2 (24.9–33.4)	1.1 (1–1.3)	–2.62 (–2.68 – –2.56)

Latin America and Caribbean	7.1 (6.8–7.6)	2.6 (2.5–2.8)	6.4 (6.1–6.8)	1.8 (1.7–1.9)	5.7 (5.4–6)	1.2 (1.1–1.3)	5.4 (4.7–5.8)	0.9 (0.8–0.9)	–3.63 (–3.69 – –3.55)

North Africa and Middle East	11.6 (8–15.3)	4.9 (3.3–6.9)	10 (7.7–12.8)	3.4 (2.6–4.4)	9.1 (7.8–10.7)	2.4 (2.1–2.9)	8.9 (7.5–10.6)	1.9 (1.6–2.2)	–3.05 (–3.09 – –3.02)

Sub-Saharan Africa	16.9 (13.1–20.8)	6.7 (5.2–8.3)	15.8 (12.6–19.2)	5 (4–6)	14.6 (12.5–17.5)	3.7 (3.2–4.4)	14.9 (12.6–18.2)	2.8 (2.5–3.5)	–2.83 (–2.84 – –2.81)

**By Country**									

India	142.6 (109.7–195.4)	27.5 (21–38)	156.3 (119.2–209.9)	22.6 (17–30.9)	175 (133.8–242.8)	19.4 (14.6–27.5)	166 (137.5–219.7)	14.3 (11.8–19)	–2.18 (–2.26 – –2.1)

Pakistan	16.6 (12.1–24.4)	26.9 (19.4–40.1)	21.8 (16.2–31)	28.2 (20.8–42.2)	22.5 (16.8–31.7)	22.4 (16.5–32.9)	24.2 (17.9–34.5)	17.9 (13.1–25.8)	–1.35 (–1.38 – –1.32)

Bangladesh	15.3 (10.1–23.9)	28.2 (18.2–44.3)	15.7 (10.5–25.1)	26.1 (17.5–40.8)	17.6 (12.3–28.2)	20.6 (14.4–32.9)	20.9 (14.1–36)	16.3 (11.1–27.9)	–1.81 (–1.9 – –1.71)

Nepal	3.3 (2.2–5.4)	31.7 (20.4–52.5)	3.1 (2–4.9)	23.1 (14.4–38.1)	3.4 (2.3–5.4)	18.9 (12.7–31)	3.8 (2.6–5.9)	17 (11.5–26.8)	–2.05 (–2.07 – –2.01)

Bhutan	0.1 (0.1–0.1)	30.2 (19.2–51.2)	0.1 (0.1–0.2)	23.1 (14.8–43.8)	0.1 (0.1–0.2)	17.8 (11.3–37.2)	0.1 (0–0.2)	14.3 (8–27.4)	–2.48 (–2.52 – –2.44)


UI: uncertainty interval, CI: confidence interval, AAPC: average annual percentage change.

**Figure 2:**
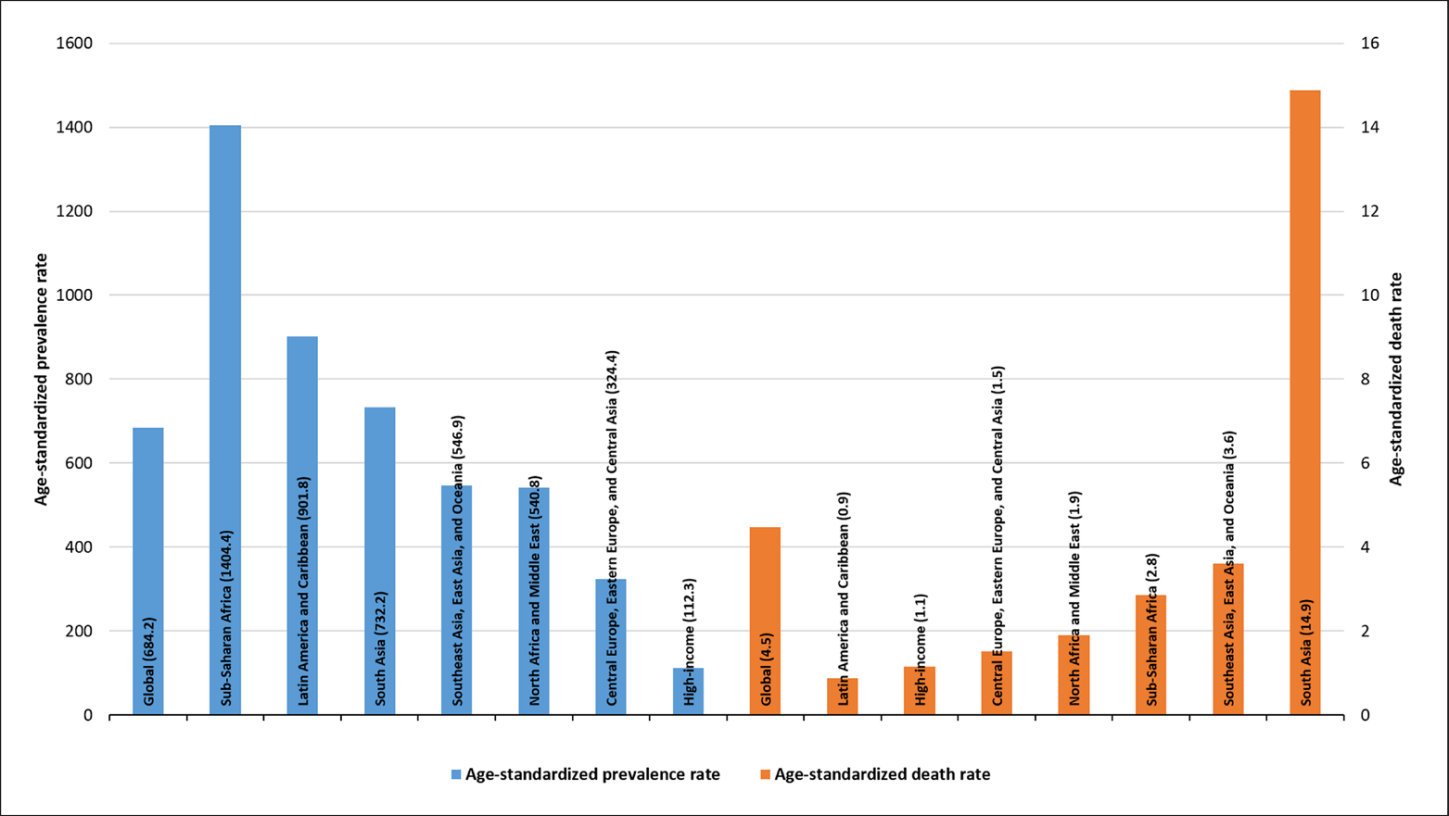
The distribution of prevalence and deaths at global and regional level for the year 2021.

The age standardized prevalence rate of RHD showed significant increase in three regions; the North Africa and Middle East region with and AAPC of 0.17 (95% CI: 0.16 to 0.17) followed by South Asia with AAPC of 0.12 (95%:0.11 to 0.13) and Sub-Saharan Africa with AAPC of 0.11 (95% CI: 0.11 to 0.11) (Table 4). According to the estimated for the year 2021, Sub-Saharan African region contributed to 28.3%, South Asian region contributed to the 26.2%, and Southeast Asia, East Asia, and Oceanian region contributed to 22.7% of the global prevalent cases of RHD. While, South Asian region contributed to 57.6% of the global deaths due to RHD followed by Southeast Asia, East Asia, and Oceanian region with the contribution of 24.0%, (Figure 2).

**Table 4:** The trend analysis of rheumatic heart disease age-standardized prevalence, DALYs, and death rate in last three decades at global and regional level.


	GLOBAL	SOUTH ASIA	SOUTHEAST ASIA, EAST ASIA, AND OCEANIA	CENTRAL EUROPE, EASTERN EUROPE, AND CENTRAL ASIA	HIGH-INCOME	LATIN AMERICA AND CARIBBEAN	NORTH AFRICA AND MIDDLE EAST	SUB-SAHARAN AFRICA

**Age-standardized prevalence rate: AAPC [95% CI]**								

Full Range	0.4 [0.39–0.4]	0.12 [0.11–0.13]	–0.4 [–0.41 – –0.38]	0.05 [0.04–0.05]	–0.07 [–0.1 – –0.04]	–0.02 [–0.02 – –0.01]	0.17 [0.16–0.17]	0.11 [0.11–0.11]

1991–2001	0.04 [0.03–0.06]	0.08 [0.06–0.1]	–1.3 [–1.34 – –1.25]	–0.28 [–0.3 – –0.26]	0.37 [0.31–0.44]	–0.06 [–0.07 – –0.05]	0.34 [0.32–0.35]	0.07 [0.07–0.08]

2002–2011	0.73 [0.71–0.75]	0.33 [0.3–0.34]	0.42 [0.39–0.46]	0.26 [0.24–0.28]	–0.75 [–0.81 – –0.71]	–0.07 [–0.08 – –0.07]	0.21 [0.19–0.22]	0.23 [0.22–0.23]

2012–2021	0.43 [0.41–0.44]	–0.09 [–0.12 – –0.06]	–0.29 [–0.33 – –0.26]	0.26 [0.24–0.28]	0.39 [0.3–0.46]	0.11 [0.1–0.11]	–0.03 [–0.05 – –0.02]	0.01 [0–0.01]

**Age-standardized DALYs rate: AAPC [95% CI]**								

Full Range	–2.47 [–2.49 – –2.44]	–2.22 [–2.27 – –2.17]	–4.22 [–4.26 – –4.17]	–4.01 [–4.09 – –3.94]	–2.8 [–2.85 – –2.75]	–2.1 [–2.13 – –2.05]	–2.94 [–2.97 – –2.91]	–1.98 [–1.99 – –1.97]

1991–2001	–2.52 [–2.59 – –2.43]	–1.59 [–1.7 – –1.47]	–4.71 [–4.83 – –4.55]	–3.57 [–3.72 – –3.39]	–4.04 [–4.16 – –3.92]	–2.59 [–2.68 – –2.47]	–3.3 [–3.39 – –3.23]	–2.21 [–2.24 – –2.19]

2002–2011	–2.51 [–2.56 – –2.44]	–1.88 [–1.99 – –1.71]	–4.83 [–4.94 – –4.76]	–5.32 [–5.43 – –5.12]	–3 [–3.1 – –2.88]	–2.17 [–2.3 – –2.08]	–2.96 [–3.05 – –2.88]	–2.11 [–2.14 – –2.09]

2012–2021	–2.35 [–2.39 – –2.3]	–3.06 [–3.24 – –2.94]	–3.12 [–3.24 – –3]	–3.13 [–3.37 – –2.94]	–1.19 [–1.36 – –1.03]	–1.19 [–1.3 – –1.05]	–2.53 [–2.62 – –2.42]	–1.59 [–1.62 – –1.56]

**Age-standardized death rate: AAPC [95% CI]**								

Full Range	–2.66 [–2.7 – –2.63]	–2.06 [–2.12 – –1.99]	–4.54 [–4.6 – –4.48]	–4.32 [–4.41 – –4.23]	–2.57 [–2.63 – –2.49]	–3.6 [–3.66 – –3.52]	–3.05 [–3.09 – –3.02]	–2.83 [–2.84 – –2.81]

1991–2001	–2.64 [–2.72 – –2.53]	–1.74 [–1.98 – –1.54]	–4.6 [–4.74 – –4.45]	–3.69 [–3.92 – –3.44]	–3.49 [–3.67 – –3.25]	–3.68 [–3.86 – –3.46]	–3.31 [–3.38 – –3.22]	–2.89 [–2.92 – –2.85]

2002–2011	–2.69 [–2.75 – –2.61]	–1.37 [–1.57 – –1.22]	–5.33 [–5.5 – –5.22]	–5.29 [–5.45 – –5.07]	–2.74 [–2.89 – –2.55]	–3.79 [–4.08 – –3.61]	–3.37 [–3.45 – –3.23]	–3 [–3.03 – –2.98]

2012–2021	–2.63 [–2.74 – –2.55]	–2.98 [–3.16 – –2.82]	–3.77 [–3.94 – –3.58]	–3.84 [–4.15 – –3.65]	–1.41 [–1.57 – –1.2]	–2.84 [–3.05 – –2.53]	–2.54 [–2.66 – –2.41]	–2.54 [–2.57 – –2.51]


AAPC: average annual percentage change, CI: confidence interval, DALYs: disability-adjusted life years.

South Asian region observed to have more rapid decline in RHD related age-standardized death rate in last two decades with AAPC of –2.53 (95% CI: –2.71 to –2.26) between 2010 and 2019 and – –2.91 (95% CI: –3.19 to –2.65) between 2000 and 2009 as compared to –0.58 (95% CI: –0.97 to –0.29) between 1991 and 1921 (Table 4).

## Discussion

In recent times, there has been a renewed focus on addressing the prevention, control, and elimination of RHD, repositioning it as a significant public health concern [17]. In 2018, every member state of the World Health Organization made a collective commitment to prioritize RHD once again on the global health agenda. Consequently, it is crucial to gain a comprehensive understanding of the global prevalence of RHD and to pinpoint the specific populations, countries, and regions in need of targeted interventions and attention. The results of this study shed light on the burden of RHD at global and South Asian regional level and its trends over the past three decades. Firstly, in 2021, there were an estimated 54,785,119 cases of RHD worldwide out of which 14,378,842 were in South Asian region accounting for 26.2% of the total prevalent cases. The global age-standardized point prevalence revealed an upward trend between 1991 and 2021, with an AAPC of 0.40 (0.39 to 0.40). At the regional level, highest increase in ASR for RHD prevalence was observed for the North Africa and Middle Eastern region with an AAPC of 0.17 (0.16–0.17) followed by the South Asian region with an AAPC of 0.12 (0.11 to 0.13) and Sub-Saharan African region with AAPC of 0.11 (0.11–0.11) between 1991 and 2021. This increase suggests that despite advancements in healthcare, RHD remains a significant concern on a global scale. Among various other factors, population growth can be the main force of RHD burden known for high fertility and lower life expectancy, especially in lower low- and middle income regions [18].

Another critical aspect of this study is the mortality associated with RHD. In 2021, RHD was responsible for 373,345 deaths globally, of which 214,999 occurred in the South Asian Region, representing more than half (57.6%) of the global deaths due to RHD. The ASR of RHD-related deaths also showed a significant decrease between 1991 and 2021, globally and in the South Asian region, with AAPCs of –2.66 and –2.06, respectively. This decline in mortality is a positive sign and suggests improvements in the management and treatment of RHD but disproportionally higher contribution from South Asian region to the global mortality is alarming. These disparities can be attributed to several key factors, including the growing disparities in national economic development and income inequality. Additionally, there is a prevailing issue of underinvestment in the construction of public health systems, which is often observed in low- and middle-income countries. These shared challenges result in inadequate healthcare resources and limited technical support for RHD prevention. Furthermore, there exists a substantial disparity in the access to treatment options with favorable outcomes, particularly for advanced-stage RHD patients [19].

Notably, numerous previous studies have highlighted a notable prevalence of RHD among women, particularly those in the childbearing age group, and this trend is even more pronounced in regions with lower Socio-Demographic Index (SDI) scores as compared to more affluent areas [20,21,22]. The study further revealed that RHD accounted for a substantial burden in terms of DALYs. In 2021, globally, RHD contributed to 13,426,369 DALYs, with an ASR of 162.12 per 100,000 population. In the South Asian region, RHD resulted in 7,656,479 DALYs, with an ASR of 453.58. Notably, the ASR of DALYs exhibited a downward trend between 1991 and 2021, both globally and in the South Asian region, indicating progress in terms of reducing the disease’s impact.

The distribution and burden of RHD are influenced by three primary categories of factors. Firstly, environmental factors play a significant role, including aspects such as overcrowding, socioeconomic status, unsanitary conditions, limited access to medical care, and suboptimal nutritional status [7]. Secondly, host and genetic factors contribute to the disease’s dynamics. Lastly, the type of infecting organism also plays a role in RHD development [7].

Early diagnosis, including the use of echocardiography screening, offers a promising avenue for increasing awareness and developing prevention strategies aimed at reducing the need for medical intervention [23]. RHD remains a significant contributor to the disease burden in this region, affecting individuals during their most productive and vital life stages. Given that RHD primarily impacts the younger population, it detrimentally affects the region’s potential and productivity [14]. Therefore, it is imperative that we prioritize investments in RHD prevention and control efforts.

Healthcare professionals have a critical advocacy role to play in influencing policymakers to initiate interventions aimed at preventing and controlling RHD [14]. One of the most effective approaches for RHD prevention involves creating an enabling environment through policy interventions. This can encompass initiatives that promote sanitation, hygiene, improved living conditions, access to proper nutrition, and equitable access to affordable, high-quality healthcare [24,25].

Historically, factors contributing to the high burden of RHD in South Asia include inadequate access to healthcare, poor living conditions, and limited awareness about preventive measures [26]. Social and economic factors like rural residence have been linked to a higher prevalence of RHD in the region [27]. Policies focusing on preventing modifiable risk factors and improving access to essential medicines are crucial in reducing the burden of RHD in South Asian countries [28]. The average number of people per household and the rate of antibiotic use or access to medical care were not specifically addressed in the provided contexts. However, an increase in health facilities could potentially contribute to a decrease in RHD death rates by improving early detection and management of the disease [29].

Key strategies for RHD prevention include strengthening primary healthcare services to detect children with streptococcal pharyngitis, implementing opportunistic screening for RHD, and carrying out evidence-based primary and secondary preventive interventions. Additionally, the establishment of robust, population-based registry centers is essential for surveillance purposes, allowing for the monitoring of trends, management practices, and outcomes. This monitoring is crucial for evaluating the impact of primary preventive interventions implemented at both the community and healthcare system levels.

While this study relies on estimates generated by the GBD study using technically sound analytical methods, it’s important to acknowledge certain limitations. Firstly, the accuracy and robustness of the GBD estimates are contingent on the quality and quantity of available data. Potential bias stemming from disease miscoding and misclassification may have influenced the results. Secondly, the diagnosis and detection of RHD have evolved over time and can vary between countries, introducing another potential source of bias. Lastly, the GBD estimates are reliant on existing literature. Considering the limited research infrastructure in this region, the data used in the GBD methodology may contain errors. Consequently, any results that seem contradictory should be subject to further verification through firsthand data and experiences.

## Conclusion

The escalating trend in the age-standardized point prevalence of RHD underscores the persistent global challenge it poses, with a particularly concerning impact in South Asia. This region alone accounts for over half of the worldwide RHD-related deaths. Nevertheless, there is reason for optimism as decreasing trends in ASR of RHD-related deaths and DALYs suggest advancements in RHD management and treatment at both global and regional levels.

Despite the significant decline in ASR for RHD deaths and DALYs over the past decade, the growing population in South Asian countries has led to an increase in the number of individuals at risk. It’s imperative to concentrate efforts on low and low-middle socio-demographic index regions with the highest prevalence and death to prevent the resurgence of this disease. A special focus should be placed on the well-being of female individuals in RHD control efforts. Tailored policies and the allocation of medical resources should prioritize gender-specific considerations to effectively combat this condition.
